# A comprehensive evaluation of the therapeutic potential of silibinin: a ray of hope in cancer treatment

**DOI:** 10.3389/fphar.2024.1349745

**Published:** 2024-02-29

**Authors:** Pantha Prodip Ray, Mohammad Ashraful Islam, Mohammad Safiqul Islam, Aixia Han, Peiwu Geng, Md. Abdul Aziz, Abdullah Al Mamun

**Affiliations:** ^1^ Department of Pharmacy, State University of Bangladesh, Dhaka, Bangladesh; ^2^ Department of Pharmacy, University of Asia Pacific, Dhaka, Bangladesh; ^3^ Department of Pharmacy, Noakhali Science and Technology University, Noakhali, Bangladesh; ^4^ Key Laboratory of Joint Diagnosis and Treatment of Chronic Liver Disease and Liver Cancer of Lishui of The Sixth Affiliated Hospital of Wenzhou Medical University, Lishui People’s Hospital, Lishui, China

**Keywords:** silibinin, cancer, apoptosis, antioxidant, anti-inflammatory, anti-proliferative, anti-metastatic, anti-angiogenic

## Abstract

Natural compounds hold promise in the search for cancer therapies due to their unique chemical structures and combinations that may effectively combat cancer while minimizing toxicity and side effects compared to conventional treatments. Silibinin, a natural lignan, has been found to possess strong anti-cancer activity against several types of human cancers based on emerging research. This study aims to provide an overview of the therapeutic potential of silibinin in the treatment and prevention of cancers. A comprehensive search was conducted using various internet databases such as PubMed, Google Scholar, and ScienceDirect to identify relevant research papers. Silibinin has been shown to exhibit anticancer activity against several types of cancers, including liver, lungs, breast, prostate, colorectal, skin, and bladder cancers. Its multifaceted mechanisms of action contribute to its therapeutic effects. Silibinin exerts antioxidant, anti-inflammatory, anti-proliferative, pro-apoptotic, anti-metastatic, and anti-angiogenic activities, making it a promising candidate for cancer therapy. One of the key mechanisms underlying the anticancer effects of silibinin is its ability to modulate multiple signaling pathways involved in cancer development and progression. It can inhibit the activation of various oncogenic pathways, including PI3K/Akt, NF-κB, Wnt/β-catenin, and MAPK pathways, thereby suppressing cancer cell proliferation, inducing cell cycle arrest, and promoting apoptosis. Silibinin possesses great potential as an effective treatment agent for cancer. The multifaceted mechanisms of action, favorable safety profile, and potential synergistic effects of silibinin with conventional therapies make it an attractive candidate for further investigation and development as a cancer treatment. However, more extensive clinical studies are necessary to fully establish the efficacy, optimal dosage, and long-term effects of silibinin in cancer treatment.

## 1 Introduction

Silibinin is a flavonolignan derived from milk thistle (*Silybum marianum*) that possesses antioxidant, antineoplastic, and hepatoprotective properties. It can be classified as a polyphenol, an aromatic ether, a benzodioxine, and a secondary alpha-hydroxy ketone. It is the primary active component of silymarin, an extract from milk thistle seeds, which contains a mixture of flavonolignans including silibinin, isosilibinin, silicristin, silidianin, and others (Kaipa et al., 2020; Verdura et al., 2021).

Silibinin exists as a combination of two diastereomers, silybin A and silybin B, present in roughly equal proportions. *In vitro* and animal studies indicate that silibinin possesses hepatoprotective properties, offering protection to liver cells against toxins. It has also shown *in vitro* anti-cancer effects against several types of cancer cells, including prostate adenocarcinoma, breast carcinoma (both estrogen-dependent and -independent), ectocervical carcinoma, colon cancer, and small and non-small lung carcinoma cells (García-Maceira and Mateo, 2009; Verdura et al., 2021).

**FIGURE 1:**
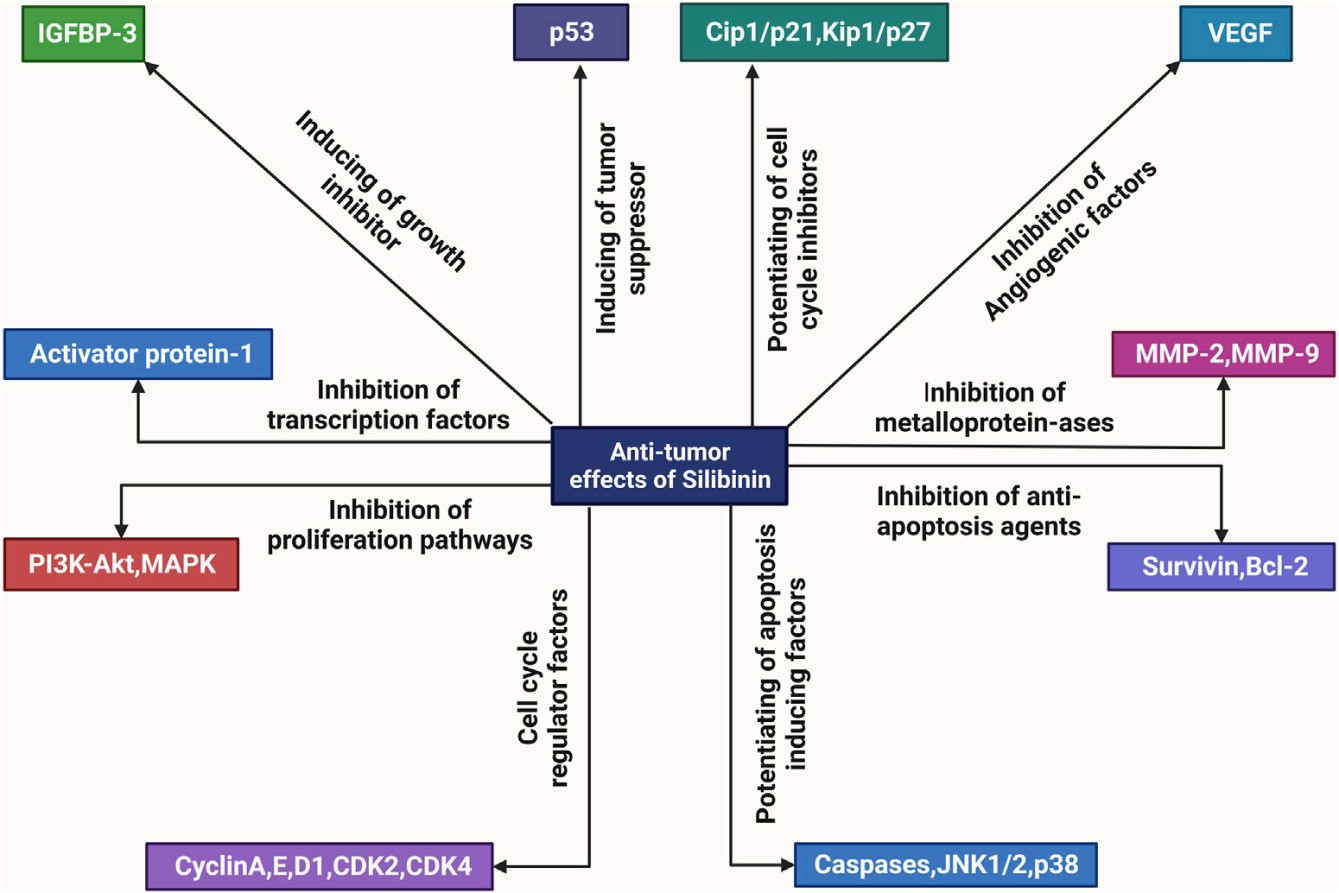
An overview of the molecular mechanisms underlying the anti-tumor actions of silibinin. Silibinin exerts a direct inhibitory effect on various factors involved in cell division and proliferation, including metalloproteinases (MMPs), anti-apoptosis proteins such as Bcl-2, growth factors such as Flt, VEGF, AR, PSA, and IGF, cell mitogen controllers such as PI3K, Akt, MAPK, cell cycle regulators such as the CDK family and pSTAT3. Additionally, it enhances the activity of apoptotic inducers such as JNK (c-Jun N-terminal kinases), Cip and Kip families, and growth inhibitors such as IGFBP. Consequently, silibinin exhibits anti-tumor effects.

Bioactive silibinin is increasingly recognized for its potential as a groundbreaking anti-cancer therapeutic strategy, (Figure 1), besides other activities such as hepatoprotection (Sahibzada et al., 2017, 2021). Within the landscape of cancer chemotherapy, the application of either naturally occurring or synthetically produced chemical substances is commonplace, with the aim of preventing, inhibiting, or reversing the recurrence and progression of cancer. Silymarin, specifically in its silybin-enriched form, stands out as a particularly intriguing natural remedy. Silibinin exhibits a diverse array of inhibitory effects on various cancer cells, encompassing growth suppression, anti-inflammatory properties, regulation of the cell cycle, induction of apoptosis, chemo-sensitization, inhibition of angiogenesis, reversal of multi-drug resistance, and prevention of invasion and metastasis. Its capacity to target fundamental cancer hallmarks, including heightened angiogenic and invasive tendencies, self-sufficiency in growth signals, insensitivity to antigrowth signals, and evasion of apoptosis, positions silibinin as an efficacious agent in both cancer chemotherapy and chemoprevention (Ting et al., 2013; Polachi et al., 2016; Sahibzada et al., 2017; Aziz et al., 2021; Sahibzada et al., 2021).

The primary objective of this study is to conduct a comprehensive review and synthesis of the mechanistic underpinnings of silibinin across different cancer types. This entails a thorough examination and summarization of the intricate molecular processes through which silibinin exerts its potential influence on cancer cells.

## 2 Role of silibinin in cancer

### 2.1 Mechanism of action of silibinin in lung cancer

Lung cancer is divided into two main types: small-cell lung cancer (SCLC) and non-small cell lung cancer (NSCLC) (Zhu et al., 2016). SCLC accounts for approximately 15% of all lung cancer cases and is characterized by high sensitivity to chemotherapy, early spread of the disease, rapid tumor growth, and strong association with tobacco smoking (Romanidou et al., 2016). In elderly patients, especially heavy smokers with cardiovascular and pulmonary comorbidities, SCLC often presents with rapid onset symptoms due to paraneoplastic syndrome and intrathoracic tumor growth. The standard first-line treatment for metastatic SCLC is platinum-based chemotherapy combined with etoposide (Fennell and De Ruysscher, 2011).

**FIGURE 2:**
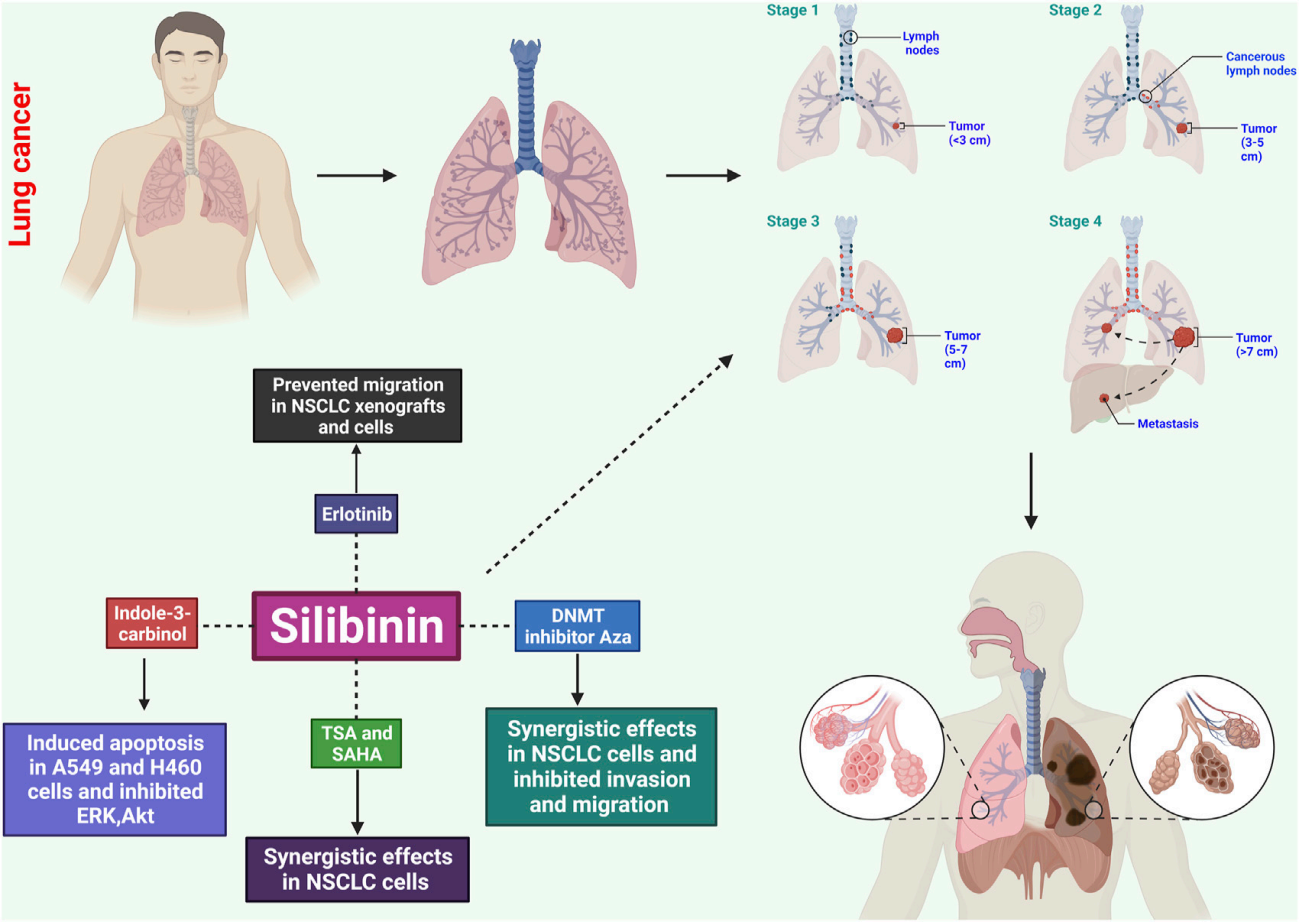
Potential effects of silibinin on lung cancer through several mechanisms. These include causing apoptosis, disrupting the cell cycle, inhibiting angiogenesis, exhibiting anti-inflammatory effects, inhibiting metastatic spread, and influencing signaling pathways. These mechanisms help control the growth and survival of cancerous cells, prevent uncontrolled growth, and limit blood supply to tumors.

Silibinin has shown significant effects on the growth inhibition and induction of apoptotic cell death in both small and non-small human lung carcinoma cells (Figure 2) (Sharma et al., 2003). In NSCLC cells, silibinin inhibits the activity of histone deacetylase (HDAC). The study examined the time-dependent and concentration-dependent inhibitory effects of silibinin on HDAC activity, revealing its potential to modulate HDAC activity. While trichostatin A (TSA) exhibited stronger inhibition of HDAC activity, silibinin showed a more prolonged inhibition. Silibinin also led to an increase in global acetylation of histone H4 and H3 in cellular chromatin. In addition to HDAC activity, silibinin affected downstream substrates involved in gene expression, such as acetylated histone H3 and H4 (Mateen et al., 2012).

Further analysis using quantitative real-time polymerase chain reaction (qRT-PCR), Western blotting, invasion assay, wound healing assay, and *in vitro* angiogenesis revealed that silibinin exhibited concentration-dependent inhibition of cell proliferation in A549, H460, and H492 cells. This was accompanied by G0/G1 cell cycle arrest, inhibition of tumor angiogenesis, apoptosis, invasion, migration, and suppression of tumor sphere formation. Silibinin downregulated phosphorylated epidermal growth factor receptor (EGFR) expression and inhibited downstream signaling pathways, including JAK2/STAT5 and PI3K/AKT. This resulted in reduced expression of matrix metalloproteinase, PD-L1, and vascular endothelial growth factor. Silibinin also disrupted the STAT5/PD-L1 complex by binding to the PD-L1 promoter region, suggesting its potential as a tumor immunotherapy and cancer stem cell-targeted therapy (Rugamba et al., 2021).

### 2.2 Mechanism of action of silibinin in breast cancer

Breast cancer is a significant cause of death in women globally, and although various chemotherapeutics have been developed for its treatment, concerns regarding low survival rates and high recurrence after chemotherapy and radiation persist. In order to enhance the effectiveness of breast cancer therapy, researchers investigated the antiproliferative effects of a combination of two herbal compounds, chrysin and silibinin, in T47D breast cancer cells (Maasomi et al., 2017). Both chrysin and silibinin individually reduced cell proliferation in a dose- and time-dependent manner, and their combined administration synergistically suppressed growth (Maasomi et al., 2017). Using the median-effect approach, the researchers determined that the combination treatments exhibited combination indices (CI) of 1, indicating synergism in inhibiting T47D cell growth (Maasomi et al., 2017). Furthermore, after 48 h of treatment, qPCR analysis revealed that the combination of medications synergistically reduced the mRNA levels of hTERT and cyclin D1 compared to the use of the individual medicines alone (*p* = 0.05). These findings suggest that the synergistic antiproliferative effects of chrysin and silibinin are associated with the downregulation of cyclin D1 and hTERT genes, indicating the potential usefulness of their combination in breast cancer treatment (Maasomi et al., 2017).

**FIGURE 3:**
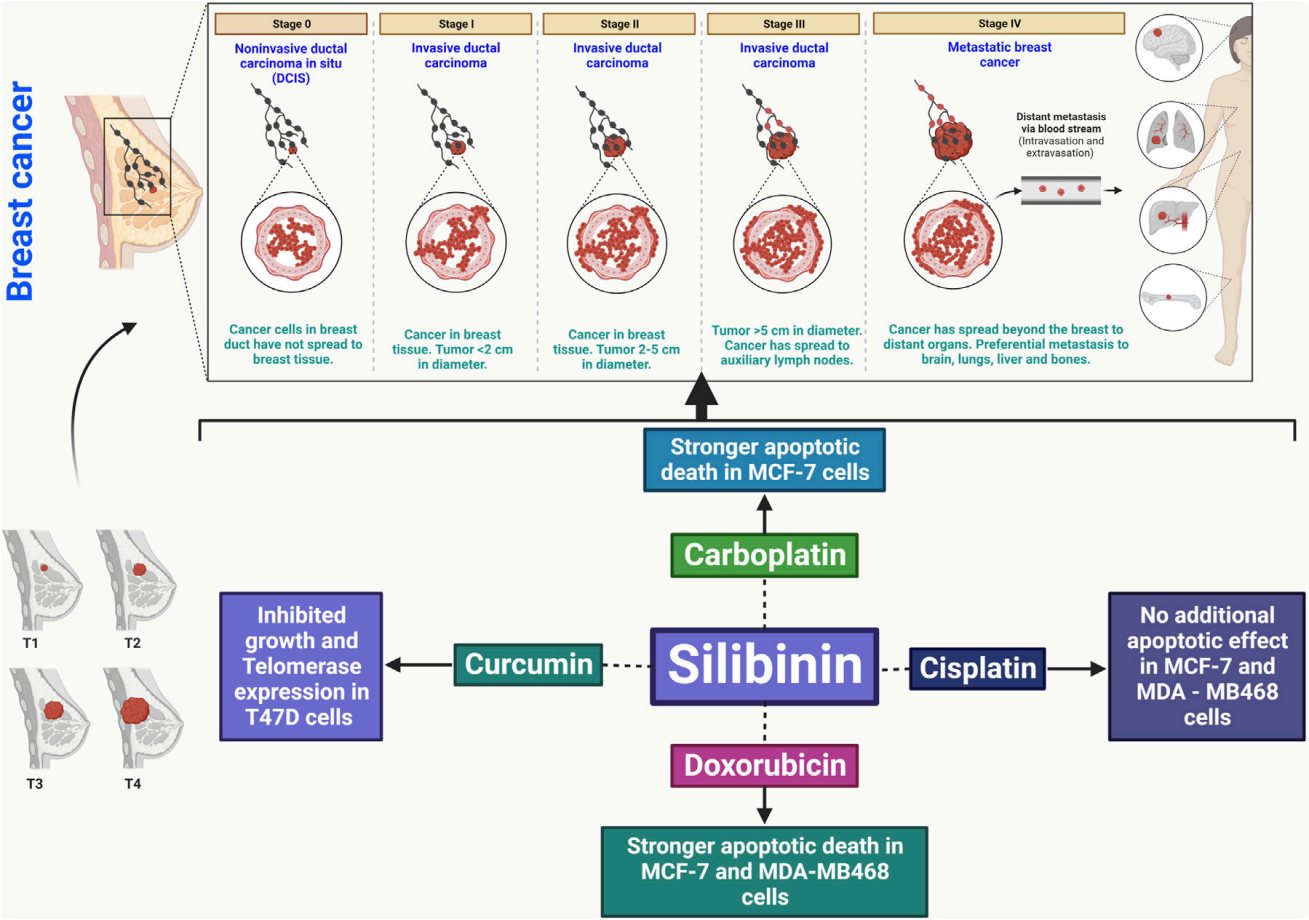
Silibinin has the potential to treat breast cancer by promoting apoptosis, disrupting the cell cycle, inhibiting cancer cell division, and reducing angiogenic factors. It also has anti-inflammatory effects and inhibits metastasis, interacting with key signaling pathways.

Silibinin shows promise as an improvement in breast cancer treatment, particularly when combined with chemotherapy drugs such as carboplatin, cisplatin, and doxorubicin, as it exhibits a synergistic effect against cancer cells (Binienda et al., 2020). This combination therapy may be beneficial for the treatment of aggressive forms of breast carcinoma. Silibinin inhibits proliferation, induces apoptosis, and inhibits metastasis through various mechanisms (Figure 3) (Binienda et al., 2020). It is feasible to combine silibinin with different anticancer medications, offering a potential treatment option for individuals with breast cancer (Binienda et al., 2020).

In various cancer models, silibinin, a naturally occurring chemical with no harmful effects, demonstrates anticancer properties and has the potential to enhance the lethal effects of chemotherapy. The effects of silibinin on enhancing sensitivity were studied in human breast cell lines resistant to doxorubicin (DOX) and paclitaxel (PAC) (Molavi et al., 2017). Silibinin inhibited cell proliferation in different cell lines, with IC_50_ values ranging from 200 to 570 μM. In DOX-resistant MDA-MB-435 cells, treatment with 200 μM silibinin significantly reduced the DOX IC_50_ from 71 to 10 g/mL and effectively suppressed key oncogenic pathways such as STAT3, AKT, and ERK. Furthermore, when DOX-resistant MDA-MB-435 cells were treated with silibinin at a concentration of 400 μM for 48 h, there was a 50% reduction in colony numbers compared to DMSO-treated cells. Combining silibinin at a concentration of 400 μM with PAC at a concentration of 250 nM resulted in synergistic effects in PAC-resistant MCF-7 cells (CI = 0.81). Overall, silibinin can be utilized to treat breast cancer by sensitizing chemo-resistant cells to chemotherapy drugs (Molavi et al., 2017).

Silibinin-induced apoptosis in MCF-7 cells was enhanced by an estrogen receptor (ER) antagonist through the upregulation of caspase 6, which was a result of suppressing ER signaling. Additionally, the upregulation of autophagy induced by silibinin contributed to the aggravation of apoptosis, and this effect was further amplified by ER inhibition. Silibinin was found to inhibit the expression of Akt/mTOR and extracellular signal-related kinase (ERK), which are involved in autophagy and apoptosis, respectively. Co-treatment with an ER inhibitor further enhanced these effects. Silibinin administration to ER-positive MCF-7 cells suppressed the expression of ER, resulting in the inhibition of the mTOR and ERK signaling pathways downstream of ER, leading to autophagy and cell death (Zheng et al., 2015).

Silibinin has been discovered to restrict the activation of AP-1 through MAPK signaling pathways, thereby inhibiting PMA-induced MMP-9 gene transcription. Moreover, experiments using Matrigel demonstrated that silibinin inhibits the invasion of MCF-7 cells triggered by PMA. These findings suggest that silibinin could serve as a potential anti-metastatic drug by inhibiting AP-1-dependent MMP-9 gene expression and thereby suppressing PMA-induced cancer cell invasion (Lee et al., 2007). In MCF-7 breast cancer cells, researchers explored the effects of silibinin, a natural flavonoid, on miRNA expression with the aim of identifying therapeutic targets. Silibinin promoted apoptosis in a dose- and time-dependent manner in MCF-7 cells. Treatment with silibinin resulted in a reduction in miR-21 and miR-155 expression levels compared to untreated cells, as observed through qRT-PCR analysis. In silico investigation indicated potential targets for miR-21 and miR-155 within apoptotic pathways, including CASP-9, BID, APAF-1, CASP-3, CASP-8, and PDCD4. Following 48 h of silibinin therapy, qRT-PCR analysis revealed the overexpression of some of these putative targets, including caspase-9 (CASP-9) and BID. Furthermore, silibinin induces apoptosis through both extrinsic and intrinsic mechanisms, as evidenced by the upregulation of CASP-9 and BID (Zadeh et al., 2016).

A study investigated the effect of silibinin on CD44 expression induced by epidermal growth factor (EGF) ligand in human breast cancer cells. EGF and TGF- significantly increased CD44 mRNA and protein expression in SKBR3 and BT474 breast cancer cells. However, treatment with EGFR inhibitors AG1478 and lapatinib inhibited the EGF ligand-induced CD44 expression. Surprisingly, silibinin treatment dose-dependently inhibited the EGF ligand-induced CD44 and matrix metalloproteinase-9 (MMP-9) expression. Silibinin also inhibited the phosphorylation of EGFR and extracellular signal-regulated kinase 1/2 (ERK1/2), a downstream signaling protein of EGFR, in response to EGF. These findings suggest that silibinin inhibits the EGFR signaling pathway and could potentially be used as a therapeutic agent to prevent breast cancer metastasis (Kim et al., 2011).

Another study evaluated the impact of silibinin on the MDA-MB-231 and MCF-7 human breast cancer cell lines in both monolayer and spheroid cultures. The half maximum inhibitory concentration (IC_50_) of silibinin in MDA-MB-231 and MCF-7 cells was determined to be 100 μM/mL at 24, 48, and 72 h of incubation. Treatment with silibinin in both monolayer and spheroid cultures resulted in increased levels of active poly-ADP-ribose polymerase and terminal deoxynucleotidyl transferase dUTP nick and labeling (TUNEL) positive cells. The number of dead cells was higher in the silibinin-treated MDA-MB-231 and MCF-7 cell lines compared to the control group. Furthermore, silibinin treatment increased the population of TUNEL positive cells and active poly-ADP-ribose polymerase in both monolayer and spheroid cultures. These findings suggest that silibinin induces cell death in breast cancer cells, both in monolayer and spheroid cultures (Ozgöçmen et al., 2020).

### 2.3 Mechanism of action of silibinin in bladder cancer

Silibinin has shown potential in the prevention and treatment of bladder tumors. In a recent study, the cytotoxic and toxicogenic effects of silibinin were investigated in bladder cancer cells with different TP53 statuses. The bladder urothelial carcinoma cell lines T24 (wild-type TP53 gene) and RT4 (mutated TP53 gene) were used in the experiments. Various parameters such as cell proliferation, clonogenic survival, apoptotic rates, genotoxicity, and the expression profiles of FRAP/mTOR, FGFR3, AKT2, DNMT1, miR100, and miR203 genes were evaluated. Silibinin demonstrated reduced proliferation and increased late apoptosis in TP53-mutated cells. Both RT4 and T24 cell lines exhibited elevated early apoptotic rates, primary DNA damage, and decreased cell colony formation in the clonogenic survival assay. Downregulation of FRAP/mTOR, AKT2, FGFR3, DNMT1, and miR100 expression was observed in RT4 cells, while miR203 modulation was detected in both cell lines. The toxicogenomic effects of silibinin on FRAP/mTOR, AKT2, FGFR3, DNMT1, and miR100 expression were found to be dependent on the TP53 status. These findings support the antiproliferative properties of silibinin, which are associated with TP53 expression (de Oliveira et al., 2017).

The study aimed to investigate the mechanisms underlying silibinin-induced apoptosis in RT4 cells with intact p53. Silibinin treatment increased the levels of p53 protein and its phosphorylation at serine 15, leading to the activation of the caspase cascade and Bid cleavage, ultimately resulting in apoptosis. The activation of p53 by silibinin was mediated through the ATM-Chk2 pathway, which subsequently triggered caspase 2-mediated apoptosis. Silibinin also facilitated the permeabilization of the mitochondrial membrane and the release of cytochrome c into the cytoplasm by promoting the rapid translocation of p53 and Bid into the mitochondria. JNK1/2 activation was identified as a link between p53-mediated caspase 2 activation and JNK1/2 activation. Additionally, silibinin-induced apoptosis involved caspase-mediated cleavage of Cip1/p21, which was inhibited by Cip1/p21 siRNA. These findings highlight the unique pathways through which silibinin induces apoptosis, involving p53-caspase 2 activation and caspase-mediated cleavage of Cip1/p21 (Tyagi et al., 2006).

Chemoprevention is emerging as a promising treatment approach for bladder cancer, a highly recurrent type of cancer. Silibinin, when tested *in vitro* using human bladder transitional cell papilloma RT4 cells, demonstrated effectiveness. Further studies were conducted on RT4 tumor xenografts to investigate the anticancer effects and underlying mechanisms of silibinin. The administration of silibinin resulted in a significant slowdown of tumor xenograft growth without any evident side effects. Tumor volume and weight were reduced by 51%–58% (*p* ≤ 0.01) and 44%–49% (*p* < 0.05), respectively. Silibinin also exhibited substantial reductions in cell proliferation and microvessel density (*p* < 0.001) and significantly enhanced apoptosis (*p* < 0.001) in the malignancies (Berthold et al., 2008).

In a study using a highly metastatic T24-L cell model, silibinin treatment was found to inhibit cell migration and invasion *in vitro* and reduce bladder cancer lung metastasis while improving animal survival *in vivo*. Silibinin achieved this by inhibiting glycogen synthase kinase-3β phosphorylation, β-catenin nuclear translocation and transactivation, and ZEB1 gene transcription, which in turn regulated the production of cytokeratins, vimentin, and matrix metalloproteinase-2 (MMP2) to reverse epithelial-mesenchymal transition (EMT). Silibinin also demonstrated a reduction in cancer stem cell (CSC) capabilities, including decreased spheroid colony formation, side population, and expression of the stem cell factor CD44. These findings provide insight into the unique mechanisms by which silibinin targets bladder cancer metastasis, involving the dual-blocking of EMT and stemness through the inactivation of β-catenin/ZEB1 signaling (Wu et al., 2013).

To investigate the apoptotic effects of silibinin on human bladder cancer cells, *in vitro* experiments were conducted. The study aimed to elucidate the mechanisms by which silibinin regulates apoptosis in bladder cancer and evaluate its intravesical effectiveness in preventing bladder cancer. Exposure to silibinin led to the activation of caspase-dependent and -independent apoptosis in 5,637 cells, accompanied by changes in mitochondrial membrane potential and the release of cytochrome c and AIF from mitochondria. Silibinin also inhibited survivin expression and induced AIF translocation to the nucleus. Oral administration of silibinin consistently suppressed the growth of 5,637 xenografts, accompanied by caspase-3 activation, downregulation of survivin, and increased AIF translocation. Additionally, intravesical administration of silibinin effectively reduced bladder carcinogenesis induced by MNU in rats, leading to a decrease in the incidence of superficial and invasive bladder lesions, along with its proapoptotic effects (Zeng et al., 2010).

Silibinin demonstrated a concentration- and time-dependent reduction in cell viability in Hep-2 cells. Furthermore, when combined with arsenic trioxide, silibinin exhibited a synergistic effect in killing Hep-2 cells. The cytotoxicity of silibinin in Hep-2 cells was associated with the accumulation of intracellular reactive oxygen species (ROS), which could be mitigated by the ROS scavenger NAC. Treatment with silibinin led to the loss of mitochondrial membrane potential (MMP) in Hep-2 cells, followed by the activation of caspase-3. Inhibition of caspase activity with Z-VAD-FMK attenuated the cytotoxic effects of silibinin. Additionally, silibinin reduced the expression of survivin in Hep-2 cells. Ultimately, silibinin induced apoptosis in Hep-2 cells by inducing oxidative stress and suppressing survivin expression (Yang et al., 2013).

In T24 and UM-UC-3 human bladder cancer cells, silibinin treatment at a concentration of 10 μM significantly inhibited proliferation, migration, invasion, and induced apoptosis. Silibinin exerted its effects by targeting the actin cytoskeleton and the phosphatidylinositide 3-kinase (PI3K)/Akt signaling pathways, which were found to crosstalk through RAS cascades. Silibinin also affected the levels of trimethylated histone H3 lysine 4 and acetylated H3 at the KRAS promoter. Moreover, silibinin targeted two specific long non-coding RNAs, HOTAIR and ZFAS1, both of which have been implicated in cancer development. These findings suggest that silibinin inhibits the progression of bladder cancer by downregulating the actin cytoskeleton and the PI3K/Akt pathways (Imai-Sumida et al., 2017).

### 2.4 Mechanism of action of silibinin in hepatocellular carcinoma

Hepatocellular carcinoma (HCC) is a recurring cancer with limited treatment options. Silibinin, known for its antihepatotoxic properties and significant preventive and anticancer effects against various epithelial malignancies, was evaluated for its efficacy against human HCC cells. Silibinin reduced the proliferation of both HepG2 and Hep3B cells, with Hep3B cells exhibiting higher cytotoxicity and induction of apoptosis. Silibinin induced G1 arrest in HepG2 cells and G1 and G2-M arrest in Hep3B cells. It increased the levels of Kip1/p27 while decreasing the levels of cyclin D1, cyclin D3, cyclin E, cyclin-dependent kinase (CDK)-2, and CDK4 in both cell lines. Silibinin also lowered the protein levels of G2-M regulators in Hep3B cells and reduced the activity of CDK2, CDK4, and CDC2 kinases in HCC cells (Berthold et al., 2008).

In an *in vivo* study, the inhibitory effects of silibinin combined with doxorubicin were investigated in hepatocellular cancer. The results showed that silibinin enhanced doxorubicin-induced growth inhibition, G2-M arrest, and cell death in HEPG2 cells. The combination of silibinin and doxorubicin reduced cdc2/p34 kinase activity when histone H1 was used as a substrate. Upstream kinases associated with cdc25C-cyclin B1-cdc2/p34 were also significantly increased by the combined treatment. Simultaneous therapy with silibinin and doxorubicin resulted in a significant increase in apoptotic cell death compared to using silibinin or doxorubicin alone. In an orthotropic rat model, the silibinin-doxorubicin combination therapy reduced tumor growth by approximately 30% at a lower dose compared to individual treatments. These findings suggest that the combination of silibinin and doxorubicin may be more effective in treating HCC patients (Li and Wang, 2016).

The antiproliferative effects of gefitinib, sorafenib, and silibinin on HCC cells were found to be dose-dependent. Combining gefitinib with silibinin enhanced the growth inhibitory effects of gefitinib in several HCC cell lines. In particular, the combination had a synergistic effect in the SNU761 cell line and an additive effect in the Huh-BAT cell line. Inhibition of EGFR-dependent Akt signaling may contribute to the combined effect. Similarly, combining silibinin with sorafenib also resulted in enhanced growth-inhibitory effects in HCC cells. Therefore, the combination of silibinin with either sorafenib or gefitinib holds potential as a therapeutic approach for HCC (Gu et al., 2015).

Combined therapy with sorafenib and silibinin effectively targets both hepatocellular carcinoma (HCC) cells and cancer stem cells by enhancing suppression of STAT3/ERK/AKT phosphorylation. The combination of silibinin and sorafenib demonstrates potent suppression of HCC cell growth and induces significant apoptosis in various HCC cell lines. In an HCC subcutaneous transplanted tumor model, the combination therapy significantly reduces tumor development compared to monotherapy. The combination treatment inhibits the phosphorylation of AKT and STAT3, as well as the expression of antiapoptotic proteins Mcl-1 and Bcl-2, leading to cancer cell death. Moreover, the combination of silibinin and sorafenib suppresses the expression of stemness-related proteins Nanog and Klf4, thereby hindering the generation and self-renewal of HCC stem cells. These findings highlight the ability of silibinin to enhance the effectiveness of sorafenib in HCC treatment, offering a potential therapeutic approach for HCC patients (Mao et al., 2018).

In a study investigating the effects of silibinin on HepG2 (human hepatocellular liver carcinoma) and HUVEC (human umbilical vein endothelial) cell lines, the efficacy of silibinin against these cell lines was examined. Different concentrations of silibinin were incubated with HepG2 and HUVEC cells for varying durations. The viability of the cells and the mode of cell death induced by silibinin were assessed using various assays. The results demonstrate that silibinin reduces the viability of HepG2 and HUVEC cells in a dose-dependent manner. HepG2 cells exhibit more consistent and dose-dependent cytotoxicity than HUVEC cells, which initially show some resistance. The mechanism of cell death appears to differ between the 2 cell lines, with HepG2 cells showing a preference for apoptosis while HUVEC cells favor necrosis (Zahir et al., 2018).

*Silybum marianum* whole extract, silymarin, and silibinin have been found to inhibit hepatocarcinogenesis and the growth of hepatocellular carcinoma (HCC) by regulating the HGF/c-Met, Wnt/β-catenin, and PI3K/Akt/mTOR signaling pathways. *In vitro* studies on HCC cell lines (HepG2 and Huh7) have confirmed their anticancer properties. In an animal study using Wistar rats treated with diethylnitrosamine (DEN)/2-acetylaminofluorene (AAF)/carbon tetrachloride (CCl4), administration of Silybum marianum whole extract, silymarin, and silibinin slowed the progression of malignant lesions. These treatments suppressed the HGF/c-Met, Wnt/β-catenin, and PI3K/Akt/mTOR signaling pathways and reduced Ki-67 expression. Furthermore, they improved liver function indicators, tumor markers, and antioxidant defense mechanisms, as indicated by increased total protein and albumin levels, decreased hepatic lipid peroxide generation, increased hepatic glutathione levels, and activation of hepatic antioxidant enzymes (Yassin et al., 2022).

In a study using nude mice with xenografts of human hepatocellular carcinoma (HuH7), silibinin demonstrated a dose-dependent reduction of HuH7 xenografts. This reduction was accompanied by decreased production of Ki-67, alpha-fetoprotein, nuclear NF-kappa B content, polo-like kinase 1, and phosphorylated retinoblastoma protein (Rb), as well as reduced expression of the E2F1/DP1 complex. Conversely, silibinin increased the expression of the p27/CDK4 complex and checkpoint kinase 1. Apoptosis induced by silibinin in HuH7 xenografts was associated with inhibition of survivin phosphorylation. Silibinin also reduced phosphorylated Akt (p-Akt) levels through decreased p-ERK and increased PTEN expression, indicating the involvement of the PTEN/PI3K/Akt and ERK pathways in the anti-HCC activities of silibinin *in vivo*. Additionally, silibinin increased the expression of acetylated histones H3 and H4, as well as superoxide dismutase 1 (SOD-1), further contributing to the inhibition of cell proliferation, cell cycle progression, and HCC xenograft growth (Cui et al., 2009).

### 2.5 Mechanism of action of silibinin in prostate cancer

The limitations of standard chemotherapy in treating advanced invasive malignancies, including prostate cancer, have underscored the need for alternative cancer control strategies. Prostate cancer, predominantly diagnosed in elderly males, is a complex disease with diverse variables and stages. Current treatment options for cancer mainly involve surgery, chemotherapy, and radiation. However, the effectiveness of chemotherapeutic drugs and radiotherapy is often hindered by their systemic toxicity. As a result, cancer chemoprevention has gained attention, focusing on the use of natural or synthetic substances alone or in combination to prevent, halt, or reverse cancer growth. Many phytochemicals have demonstrated anticancer potential while exhibiting minimal or no harm to normal cells. In the case of prostate cancer, silibinin specifically targets pathways such as EGFR, IGF-1R, and NF-κB (nuclear factor-kappa B). It also modulates cell-cycle regulators like cyclin-dependent kinases (CDKs), Cip/Kip, and cyclins to exert their anticancer effects. Silibinin inhibits the growth of human, mouse, and rat prostate cancer cells, as well as the formation of human prostate tumor xenografts in nude mice. It has also shown suppression of prostate cancer growth in the transgenic adenocarcinoma of mouse prostate (TRAMP) mouse model. Silibinin has entered phase I/II clinical trials in prostate cancer patients, and preliminary results suggest the need for further investigation in a broader patient population (Singh and Agarwal, 2006).

In a study focusing on rat prostate cancer cells, silibinin demonstrated both anti-proliferative and apoptotic actions. Rat prostate cancer cell lines were developed from primary prostate cancer in rats induced by an MNU-testosterone regimen. However, their suitability as a model for screening prostate cancer preventive and therapeutic drugs remained to be determined. The primary objective of this study was to evaluate the antiproliferative and apoptotic effects of silibinin, a major active flavonoid component of silymarin found in milk thistle, in rat prostate cancer cell lines. Silibinin exhibited significant growth inhibition and reduced cell viability in a dose- and time-dependent manner in all investigated cell lines. Treatment with silibinin induced G1 arrest in H-7 and I-8 cells after 12 and 24 h, but caused S phase arrest after 48 h. I-26 cells showed a minor S-phase arrest with silibinin treatment at all tested time points. These findings were consistent with a substantial suppression of DNA synthesis by silibinin. Moreover, silibinin induced significant apoptosis in all tested cell lines. Similar to silibinin, silymarin also inhibited growth and decreased viability in a dose- and time-dependent manner (Tyagi et al., 2002).

Silibinin exhibits pleiotropic anticancer effects against prostate cancer cells both in culture and in nude mice. It inhibits cell proliferation and disrupts cell cycle progression while suppressing mitogenic and cell survival signaling pathways, including epidermal growth factor receptor, insulin-like growth factor receptor type I, and nuclear factor kappa B signaling. Silibinin also enhances the therapeutic benefits of doxorubicin and shows potential for combination treatment. Additionally, it inhibits the production of proangiogenic factors by tumor cells, inhibits endothelial cell growth, induces apoptosis, and disrupts capillary tube formation. *In vivo* studies in nude mice demonstrate that silibinin suppresses the growth of advanced human prostate tumor xenografts. Its non-toxic nature and mechanism-based preventive effects have led to its initiation of phase I clinical trials in prostate cancer patients (Singh and Agarwal, 2004).

In an *in vitro* model examining the relationship between obesity and prostate cancer, silibinin was found to reduce the expression of proliferative signaling proteins such as COX-2, IL-6, AKT, ERK, and AR, which are elevated in obesity. Obese and overweight sera were shown to enhance prostate cancer cell proliferation and invasive potential, but silibinin was able to counteract these effects. However, it is important to note that this study had limitations, as it did not employ an *in vivo* model or a co-culture model that could better replicate the tumor microenvironment to confirm the *in vitro* findings (Sherman et al., 2020).

Silibinin primarily targets the sterol response element binding protein 1 (SREBP1) and inhibits abnormal lipid metabolism, proliferation, and the development of androgen-independence in prostate cancer cells. It significantly reduces lipid and cholesterol buildup in human prostate cancer cells, but not in non-neoplastic cells. Silibinin decreases the nuclear proteins of SREBP1 and SREBP2, as well as their target genes, in prostate cancer cells. The inhibition of AMPK prevents the silibinin-mediated decrease in nuclear SREBP1 and lipid accumulation. The significance of SREBP1 in silibinin’s regulation of prostate cancer cell proliferation, lipid accumulation, and cell cycle arrest is confirmed by using the selective SREBP inhibitor fat statin and persistent overexpression of SREBP1. Notably, silibinin blocks synthetic androgen-induced lipid accumulation and completely prevents the generation of androgen-independent prostate cancer cell clones by targeting SREBP1/2. These findings suggest that silibinin holds promise in combating prostate cancer by targeting abnormal lipogenesis through SREBP1/2 regulation (Nambiar et al., 2014).

### 2.6 Mechanism of action of silibinin in pancreatic cancer

**FIGURE 4:**
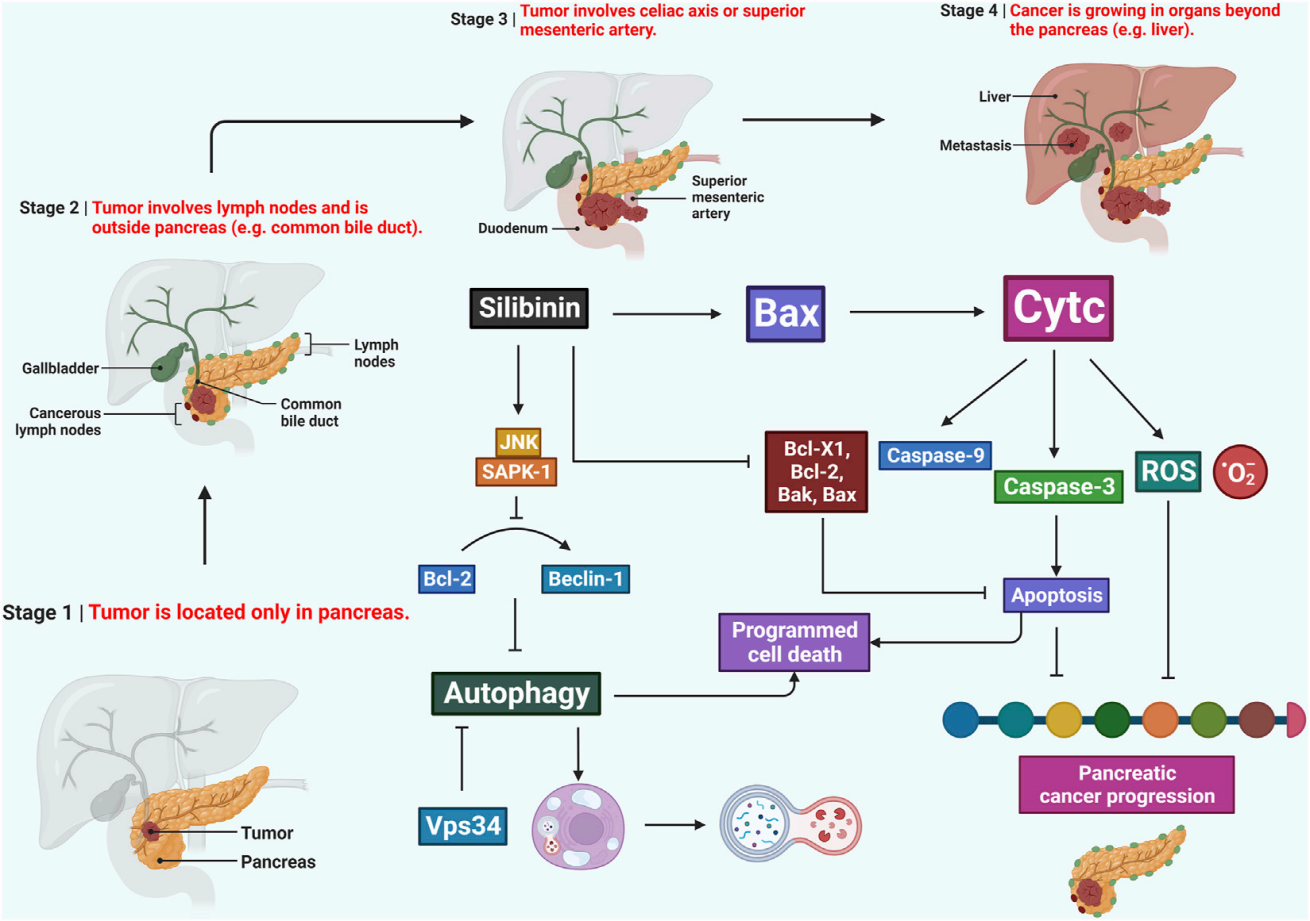
Potentiality of silibinin in combating pancreatic cancer by triggering apoptosis, disrupting the cell cycle, and modulating the inflammatory microenvironment. It also inhibits metastasis, oxidative stress, and downregulates NF-κB, making it a promising candidate for further exploration in pancreatic cancer therapeutics.

Silibinin has been shown to induce apoptosis and cell cycle arrest in human pancreatic cancer cells (Figure 4). In pancreatic cancer cells (AsPC-1, BxPC-3, and Panc-1), silibinin substantially suppresses cell growth and induces apoptosis. Silibinin alters the cell cycle in AsPC-1 cells, reducing S phase and causing G1 phase arrest, while it has no effect on the cell cycle of BxPC-3 or Panc-1 cells. These findings suggest that silibinin could be a potential chemopreventive drug in the treatment of pancreatic cancer (Ge et al., 2011).

Silibinin-mediated metabolic reprogramming has been shown to reduce cachexia and tumor development in pancreatic cancer. It inhibits pancreatic cancer cell growth and glycolytic activity in a dose-dependent manner. Metabolomics data obtained through LC-MS/MS analysis reveals that silibinin treatment induces global metabolic reprogramming in pancreatic cancer cells. Silibinin reduces the expression of c-MYC, a significant regulator of cancer metabolism, and downregulates STAT3 signaling in cancer cells. The overexpression of constitutively active STAT3 significantly reverses the silibinin-induced downregulation of c-MYC and metabolic phenotype. In an orthotopic mouse model of pancreatic cancer, silibinin inhibits tumor development, reduces proliferation, and prevents weight and muscle loss associated with cachexia. Furthermore, it enhances physical activity in tumor-bearing animals, as evidenced by improved grip strength and increased latency to fall. These findings highlight that silibinin-induced metabolic reprogramming effectively inhibits pancreatic cancer cell proliferation and attenuates cachectic characteristics in both human and animal models (Shukla et al., 2015).

Silibinin exhibits its effects on pancreatic cancer through the activation of JNK/SAPK signaling, leading to the inhibition of autophagy and mitochondrial apoptosis. This study aimed to investigate the role of silibinin in pancreatic cancer treatment. Silibinin treatment increased cell viability and apoptosis while enhancing the expression of reactive oxygen species (ROS) and ATP, both associated with mitochondrial activity. Autophagy induction was also observed in pancreatic cancer cells upon silibinin treatment. The biological effects of silibinin could be reversed by a JNK/SAPK inhibitor (Zhang et al., 2019).

Silibinin possesses pleiotropic anticancer properties, but its molecular mechanisms and effects on normal pancreatic cells are poorly understood. This research aimed to explore the impact of silibinin on human pancreatic cancer cells and normal ductal cells. Silibinin inhibited extracellular signaling-regulated kinase (ERK) and serine/threonine protein kinase (AKT) in human pancreatic cancer cells, as observed through Western blot analysis. It also increased microtubule-associated protein 1 light chain 3 (LC3B) and cleaved caspase-3 levels via c-Jun N-terminal kinase (JNK) signaling. Silibinin upregulated c-Jun, Twist-related protein 1, and Snail mRNA and protein levels while decreasing exogenous p53 levels and increasing endogenous c-Jun protein levels in pancreatic cancer cells. However, it did not affect cell viability or endogenous c-Jun levels in normal ductal cells. Silibinin increased exogenous p53 levels in normal ductal cells while reducing the expression of stemness-related genes. Furthermore, it increased Ki-67 levels in pancreatic cancer cells but decreased them in normal ductal cells (Lee et al., 2020).

Silibinin induces G1 arrest, cell death, and activates JNK/SAPK in SW1990 human pancreatic cancer cells. MTT assays revealed a dose- and time-dependent inhibitory effect of silibinin on AsPC1 and SW1990 cells. Propidium iodide staining indicated G1 phase arrest in the cell cycle, accompanied by reduced expression of cyclin D1, cyclin E2, cyclin A, and cyclin B1 as observed through Western blotting. The expression levels of G1-related cell cycle-dependent kinases, cyclin-dependent kinase (CDK)4 and CDK6, as well as p15 (p15INK4B), were decreased. Flow cytometry analysis identified early and late apoptotic cells in SW1990 cells after silibinin incubation at various doses. Silibinin increased caspase 9 and caspase 3 activity, as well as the cleavage of poly (ADP-ribose) polymerase (PARP). It decreased the expression levels of Bcl2-like 1, Bcl2-like 1 small, and Bcl2-like protein 11, while Bcl-like protein 4 expression remained unchanged, and Bcl2-like 1 small and Bcl2-like protein 11 expression levels were elevated (Zhang et al., 2018).

### 2.7 Mechanism of action of silibinin in thyroid cancer

Silibinin has been shown to inhibit TPA-induced cell migration and MMP-9 production in thyroid and breast cancer cells. The study investigated the effects of silibinin on cell migration and MMP-9 expression induced by 12-O-tetradecanoylphorbol-13-acetate (TPA). TPA significantly increased MMP-9 mRNA and protein expression in TPC-1 and MCF7 cells, while MMP-2 expression remained unaffected. Treatment with the MEK1/2 inhibitor UO126 reduced TPA-induced MMP-9 expression. Silibinin also markedly reduced TPA-induced cell migration and MMP-9 expression. Additionally, silibinin attenuated TPA-induced phosphorylation of MEK and ERK. These findings suggest that silibinin inhibits TPA-induced cell migration and MMP-9 production in thyroid and breast cancer cells through a MEK/ERK-dependent mechanism (Oh et al., 2013).

Silibinin has demonstrated potent pleiotropic anticancer effects against various human carcinoma cell types. In another study, different concentrations of silibinin (25, 50, 75, and 100 μM) were used to treat 8305c cell cultures. The metabolic activity of the treated cells was assessed using the MTT assay after 24 and 48 h. The MTT assay is a colorimetric technique used to determine cell viability and cytotoxicity. The results of the investigation revealed that silibinin exerted cytotoxic effects on the 8305c cell line. Treatment with silibinin at the specified doses led to a significant reduction in metabolic activity over time and dosage (Vahdatirad et al., 2014).

### 2.8 Mechanism of action of silibinin in cervical cancer

Cervical cancer is a significant health concern, with a high incidence and mortality rate worldwide. In 2018 alone, there were approximately 570,000 cases and 311,000 deaths attributed to cervical cancer. Silibinin has been found to inhibit the growth of cervical cancer cells by inducing G2/M cell cycle arrest through the activation of dynamin-related protein 1 (Drp1). This activation leads to mitochondrial fission failure, both *in vitro* and *in vivo*. Silibinin treatment resulted in reduced ATP content, mitochondrial membrane potential, mtDNA copy number, and reactive oxygen species levels in cervical cancer cells. It also caused excessive mitochondrial fragmentation and tubule formation. Knockdown of Drp1 prevented silibinin-induced G2/M cell cycle arrest in cervical cancer cells by inhibiting the mitochondrial fission pathway. Furthermore, silibinin exhibited a decreased proliferation of Hela cells in an *in vivo* model. This study highlights the activation of Drp1-dependent mitochondrial fission by Silibinin as a potential approach for cervical cancer prevention and therapy (You et al., 2020).

Another study investigated the combined effect of metformin, an herbal supplement, and silibinin on programmed cell death in cervical cancer cells. The study focused on C-33A cells and measured cell viability using the MTT assay and signaling protein expression through Western blot analysis. The combination of metformin and silibinin synergistically reduced cell survival in C-33A cells without affecting nonmalignant cells (HUVECs). Silibinin increased PTEN expression, while metformin elevated AMPK expression in C-33A cells. The combined treatment led to higher expression of activated caspase-3 and apoptosis-inducing factor (AIF), indicating enhanced apoptosis. This combination therapy, at a concentration that did not affect HUVECs, showed potential in inducing programmed cell death in human cervical cancer cells, suggesting a potential treatment approach for cervical cancer (Liou et al., 2015).

Silibinin has been found to suppress hypoxia-inducible factor-1 (HIF-1) accumulation and transcriptional activity in human cervical (HeLa) and hepatoma (Hep3B) cancer cells. Silibinin does not affect the degradation of HIF-1 protein or the level of HIF-1 mRNA. Instead, it inhibits HIF-1 accumulation by dephosphorylating mTOR and its downstream effectors, including ribosomal protein S6 kinase (p70S6K) and eukaryotic initiation factor 4E-binding protein-1 (4E-BP1). This pathway regulates HIF-1 production at the translational level. Silibinin also activates Akt, which is a common characteristic of mTOR inhibitors. In addition, silibinin inhibits hypoxia-induced VEGF production and demonstrates potent anticancer effects by inhibiting cell proliferation. These findings highlight the potent HIF-1 inhibitory properties of silibinin and provide insights into its mechanism of action in anticancer treatment (García-Maceira and Mateo, 2009).

Silymarin, another compound derived from milk thistle, reduces the viability of cervical cancer cells (C-33A) and induces apoptosis by activating caspase 3 and modulating Bcl-2 family proteins. Silymarin increases the expression of phosphatase and tensin homolog (PTEN), which in turn prevents Akt phosphorylation. It also inhibits C-33A cell invasion and wound healing migration in a concentration-dependent manner. Western blot analysis reveals that silymarin significantly suppresses the expression of matrix metalloproteinase-9 (MMP-9) in C-33A cells. When the PTEN gene is silenced using siRNA, the anticancer effects of silymarin are reduced. These findings demonstrate that silymarin has the ability to inhibit survival, migration, and invasion of C-33A cancer cells (Yu et al., 2012).

### 2.9 Mechanism of action of silibinin in ovarian cancer

The effect of silibinin on the SORT1 gene expression and viability of the A2780s ovarian cancer cell line were investigated. Ovarian cancer, a highly lethal gynecological tumor originating from uterine epithelial cells, often remains undetected until it metastasizes. The overexpression of the sortilin1 (SORT1) gene is observed in ovarian cancers. To assess the impact of silibinin, the A2780s ovarian cancer cell line was treated with varying concentrations of silibinin for 24 h, and the half-maximal inhibitory concentration (IC_50_) was determined. The viability of cells treated with 100 µM silibinin was evaluated at 24, 48, and 72 h. RNA was extracted after 24 and 48 h of exposure to 100 µM silibinin, followed by cDNA synthesis. The expression of the SORT1 gene was examined using real-time polymerase chain reaction (PCR) with glyceraldehyde 3-phosphate dehydrogenase (GAPDH) as the reference gene. Silibinin dose-dependently and time-dependently reduced the viability of ovarian cancer cells, accompanied by a decrease in SORT1 gene expression. These findings suggest that silibinin exhibits cytotoxic effects on the A2780s ovarian cancer cell line and may have potential as an anticancer drug (Lotfi et al., 2021).

In human ovarian clear cell carcinoma (CCC) cell lines, silibinin demonstrates its activity as a suppressor of hypoxia-inducible factor-1 (HIF-1). The CCC cell lines HAC-2, OVISE, and RMG-1 were treated with 500 µM silibinin under normoxic and hypoxic conditions for 4 h. Gene expression changes in response to hypoxia were assessed using DNA microarray, while HIF-1 expression was measured using an ELISA kit. Silibinin treatment reduced HIF-1 protein levels, as well as eIF4E2 and RPS6 mRNA expression, in HAC-2 and RMG-1 cells. Silibinin decreased HIF-1 protein under hypoxic conditions in CCC cell lines, indicating its potential as a viable anti-cancer therapy (Miyazawa et al., 2020).

Silibinin exhibits anticancer effects in human ovarian cancer cells by inhibiting epithelial-mesenchymal transition (EMT) and promoting apoptosis. The study investigated the inhibitory effects of silibinin on ovarian cancer both *in vitro* and *in vivo*. Cell viability, migration, invasion, and apoptosis were evaluated in SKOV-3 and A2870 ovarian cancer cell lines. Q-RT-PCR and Western blotting were used to assess the protein levels of signaling pathway markers. The effectiveness of silibinin in suppressing tumor growth was examined using a mouse xenograft model. *In vitro*, silibinin reversed EMT by upregulating E-cadherin expression and downregulating N-cadherin and vimentin expression. It also suppressed EMT regulators such as Snail, Slug, and ZEB1 transcription factors, and inhibited intermediate molecules including PI3K/AKT, Smad2/3, and β-catenin. *In vivo*, silibinin significantly inhibited tumor growth (Maleki et al., 2022).

The antioxidant N-acetylcysteine (NAC) was found to attenuate silibinin-induced cell death. Western blot analysis revealed that silibinin downregulated extracellular signal-regulated kinase (ERK) and Akt. Silibinin-induced cell death was hindered by the transfection of constitutively active forms of MEK and Akt. In a rat model with subcutaneous A2780 cells, silibinin treatment led to a reduction in tumor volume. Examination of tumor tissue after silibinin treatment showed a decrease in Ki-67-positive cells, an increase in transferase-mediated dUTP nick end labeling (TUNEL)-positive cells, activation of caspase-3, and suppression of p-ERK and p-Akt. Inhibition of ERK and Akt by silibinin demonstrated inhibition of tumor growth in human ovarian cancer cells. These findings suggest the potential use of silibinin as a treatment for ovarian cancer (Cho et al., 2013).

Silibinin was explored as a potential inhibitor of epidermal growth factor receptor (EGFR) tyrosine kinase for ovarian cancer treatment. EGFR is frequently upregulated and overexpressed in ovarian cancer, leading to STAT3 activation and anti-apoptotic events, resulting in resistance to platinum-based treatments. Combining EGFR targeting with platinum drugs to enhance drug sensitivity is a promising strategy in ovarian cancer treatment. Molecular simulation studies were conducted to analyze the structural and functional characteristics of silibinin as a potential EGFR kinase inhibitor, as well as its metabolic profile. The findings indicated that silibinin exhibited significant binding energy and interacted with important residues in the active site of EGFR, suggesting its potential as a therapeutic agent for ovarian cancer (Hosen et al., 2016).

### 2.10 Mechanism of action of silibinin in renal cell carcinoma

Silibinin demonstrates its ability to suppress epithelial-mesenchymal transition (EMT) in renal cell carcinoma (RCC) through autophagy-dependent Wnt/β-catenin signaling. Silibinin induces apoptosis and reduces metastasis in RCC by activating autophagy. The underlying mechanisms by which silibinin promotes autophagy are not fully understood. This study aimed to investigate the impact of silibinin on RCC metastasis *in vitro* and *in vivo*, focusing on the involvement of autophagy-dependent Wnt/β-catenin signaling. *In vitro* experiments utilized RCC 786O and ACHN cell lines, while *in vivo* studies employed RCC xenografts in nude mice. Silibinin effectively suppressed RCC metastasis and EMT both *in vitro* and *in vivo* by modulating the Wnt/β-catenin signaling pathway. Additionally, silibinin inhibited Wnt/β-catenin signaling through autophagy. The antimetastatic effects of silibinin on RCC were attributed to autophagic degradation of β-catenin induced by the drug. These findings uncover a unique mechanism by which silibinin suppresses RCC EMT and metastasis, suggesting its potential as a novel treatment option for metastatic RCC (Fan et al., 2020).

Silibinin inhibits the progression of EGFR-induced renal cell carcinoma by suppressing the EGFR/MMP-9 signaling pathway. The study examined the inhibitory effect of silibinin on EGFR-induced migration and invasion abilities of RCC cells, along with the involvement of the EGFR signaling cascade in RCC development. Silibinin effectively blocked the EGFR signal, leading to a dose-dependent reduction in RCC cell migration and invasion, particularly in EGFR-high RCC cells. Silibinin decreased the phosphorylation of EGFR and its downstream components ERK1/2, while having no effect on STAT3 or Akt phosphorylation in human RCC cell lines. The EGFR signal upregulated MMP-9 expression and activity, whereas silibinin decreased MMP-9-dependent migratory and invasive capacities of RCC cells. By blocking the EGFR/MMP-9 signaling pathway, silibinin prevented EGFR-induced migration and invasion of RCC cells, highlighting its potential as a therapeutic agent for preventing RCC metastasis (Liang et al., 2012).

Silibinin exhibits inhibitory effects on the invasion and migration of renal cancer 786-O cells *in vitro*, as well as retards xenograft development *in vivo*, and enhances the chemosensitivity to 5-fluorouracil and paclitaxel. Silibinin effectively reduces the invasion and migration of 786-O renal cell carcinoma (RCC) cells without causing cytotoxicity. To investigate the underlying mechanisms, 786-O cells were treated with silibinin at various concentrations (up to 50 μM) for a specific duration. Gelatin zymography, casein zymography, and Western blot were performed to evaluate the effects of silibinin on metalloproteinase (MMP)-2, MMP-9, urokinase plasminogen activator (u-PA), and MAPK pathway signaling proteins. Silibinin dose-dependently decreased the expressions of MMP-2, MMP-9, u-PA, p-p38, and p-Erk1/2. In cultures pre-treated with PD98059 (Erk1/2 inhibitor) and SB203580 (p38 inhibitor), lower expressions of MMP-2 and u-PA, as well as suppressed cell invasion, were observed. In an *in vivo* study using a nude mice xenograft model, solid tumors were significantly smaller 8 days after cell injection in the group receiving silibinin. By day 44, silibinin feeding resulted in a 70.1% reduction in tumor volume and a 69.7% reduction in tumor weight compared to the control group. Furthermore, combining silibinin with 5-fluorouracil, paclitaxel, vinblastine, or RAD-001 improved the chemosensitivity of 5-fluorouracil and paclitaxel. In summary, silibinin effectively suppresses the invasion and migration of 786-O cells *in vitro*, slows down xenograft development *in vivo*, and enhances the sensitivity to 5-fluorouracil and paclitaxel chemotherapy (Chang et al., 2011).

### 2.11 Mechanism of action of silibinin in liver cancer

A study aimed to develop folate-targeted nano-micelles containing silibinin as an active drug delivery method for liver cancer treatment. The researchers focused on evaluating the therapeutic efficacy of silibinin-loaded folic acid-conjugated Pluronic F127 (SLB-F127-FA) nanomicelles as a targeted drug delivery platform for liver cancer treatment. Folic acid was initially attached to the hydrophilic chains of Pluronic F127 copolymer through the Steglich esterification process to create SLB-F127-FA nanomicelles. Silibinin was then encapsulated within the self-assembled hydrophobic core of the FA-conjugated F127 to form SLB-F127-FA nanomicelles. The nanomicelles exhibited a nearly spherical shape with an average particle size of 17.7 nm. Dynamic light scattering analysis revealed that the average hydrodynamic size of non-targeted (SLB-F127) and targeted (SLB-F127-FA) nanomicelles was 19.6 nm and 29.2 nm, respectively. The SLB-F127-FA nanomicelles demonstrated a drug loading content of 2.36% and an entrapment efficiency of 79.43%. *In vitro* release studies demonstrated sustained and controlled release patterns of silibinin from the nanomicelles compared to free silibinin. The release kinetics of silibinin from SLB-F127-FA nanomicelles followed the Korsmeyer-Peppas kinetic model, indicating a dominant Fickian diffusion type release mechanism. In an *in vitro* cytotoxicity study using human liver cancer cells (HepG2), cells exposed to SLB-F127-FA nanomicelles exhibited significantly reduced viability compared to those treated with non-targeted nanomicelles (SLB-F127) or free silibinin. These findings suggest that SLB-F127-FA nanomicelles hold promise as a tailored drug delivery platform for liver cancer treatment or delivering hydrophobic drugs to tumors with folate receptor overexpression (Ghalehkhondabi et al., 2021).

The combination of silibinin and phosphatidylcholine (PC) has been developed to address the limited water solubility and poor bioavailability of silibinin. This formulation has shown improved solubility, bioavailability, and therapeutic efficacy when taken orally. Further understanding of the signaling pathways affected by silibinin is needed to fully utilize its potential in developing targeted therapeutics for liver diseases and cancer. Silibinin has demonstrated the ability to modulate various cell signaling pathways in preclinical models, with *in vitro* and *in vivo* studies indicating potential benefits against liver diseases and cancer. This study provides a summary of the pharmacokinetic features, bioavailability, safety data, clinical activity, and modulatory actions of silibinin in diverse cell signaling pathways for liver diseases and cancer (Polachi et al., 2016).

Combination therapy with sorafenib and silibinin has shown synergistic effects by inhibiting the phosphorylation of STAT3/ERK/AKT and targeting both hepatocellular carcinoma (HCC) cells and cancer stem cells. The combination of silibinin and sorafenib effectively suppressed the growth of HCC cells and induced significant apoptosis. In an HCC subcutaneous tumor model, the combination therapy inhibited tumor development more effectively compared to monotherapy. The combination treatment reduced the phosphorylation of AKT and STAT3, as well as the expression of antiapoptotic proteins like Mcl-1 and Bcl-2, leading to cancer cell death. Additionally, the combination therapy suppressed HCC stem cell development and self-renewal by downregulating the expression of stemness-related proteins such as Nanog and Klf4. These findings highlight the potential of silibinin in enhancing the effectiveness of sorafenib in HCC treatment, providing a promising therapeutic approach for HCC patients (Mao et al., 2018).

Silibinin possesses membrane-stabilizing and antioxidant properties and has been shown to promote hepatocyte regeneration, reduce inflammatory responses, and inhibit fibrogenesis in the liver. Experimental and clinical studies have provided evidence supporting these beneficial effects. In open research, long-term administration of silibinin has been found to extend the survival period of individuals with alcohol-induced liver cirrhosis. Molecular biology studies have demonstrated that silibinin can significantly reduce tumor cell growth, inhibit angiogenesis, and improve insulin resistance. These findings highlight the potential of silibinin as a therapeutic option for liver diseases and the prevention and treatment of primary liver cancer (Fehér and Lengyel, 2012).

### 2.12 Mechanism of action of silibinin in esophageal cancer

A study focused on investigating the role of AMPK signaling in mediating the anticancer effects of silibinin in esophageal squamous cell carcinoma (ESCC). The researchers examined the expression of AMPK and its involvement in ESCC, as well as the potential of silibinin to activate AMPK and limit ESCC growth. They found that AMPK was constitutively inactive in the majority of human ESCC samples. Silibinin treatment resulted in decreased ESCC cell growth *in vitro* and reduced tumorigenicity *in vivo*, without causing any adverse effects. *In vitro* experiments showed that silibinin inhibited the invasion capacity of ESCC cells and reduced the formation of lung metastases in nude mice. The anticancer effects of silibinin were found to be dependent on AMPK, as the addition of compound C (an AMPK inhibitor) or the use of shRNA against AMPK diminished the effectiveness of silibinin. Furthermore, silibinin enhanced the sensitivity of ESCC cells and tumors to the chemotherapeutic drugs 5-fluorouracil and cisplatin. These preclinical findings suggest that targeting AMPK may be a promising therapeutic approach for esophageal cancer, and silibinin could be a potential agent to activate AMPK and inhibit ESCC growth (Li et al., 2016).

### 2.13 Mechanism of action of silibinin in skin cancer

In a study, the anticancer properties of silibinin nanoparticles were evaluated using the B16 melanoma cell line. Silibinin, known for its strong antioxidant properties, has shown anticancer efficacy in various *in vivo* and *in vitro* models. Currently, it is being investigated in a phase II clinical trial for prostate cancer patients, where its safety and low toxicity in humans have been observed as significant advantages. However, the therapeutic potential of silibinin is limited due to its poor solubility and low bioavailability. To overcome this challenge, the researchers aimed to develop silibinin-loaded co-polymeric micelles and assess their ability to inhibit proliferation in B16 melanoma cells. They employed the co-solvent evaporation method to encapsulate silibinin in PEO-b-PCL and PEO-b-PBCL, resulting in polymeric micelles with an average diameter of 90 nm. The encapsulation efficiency of silibinin in the micelles was found to be 47% for PEO-b-PCL and 95% for PEO-b-PBCL. *In vitro* experiments demonstrated that polymeric micellar silibinin exhibited growth inhibitory effects comparable to free silibinin in the B16 melanoma cell line. These findings suggest that PEO-b-PBCL polymeric micelles have the potential to serve as carriers for silibinin, providing a promising approach to enhance its therapeutic efficacy by improving solubility and bioavailability (Hassankhani Rad, 2018).

In another study conducted on SKH-1 hairless mice with persistently UVB-exposed skin and tumors, the effects of silibinin on E2F transcription factors and related biological processes were investigated. UVB exposure was found to decrease the levels of E2F1 protein in the skin but increase the levels of E2F2 and E2F3 proteins. However, treatment with silibinin reversed these effects, leading to an increase in E2F1 levels and a decrease in E2F2 and E2F3 levels. The increase in E2F1 induced by silibinin was associated with the suppression of apoptosis and reductions in the levels of p53 and cyclin-dependent kinase inhibitors. On the other hand, the decrease in E2F2 and E2F3 levels caused by silibinin was accompanied by reductions in the levels of cyclin-dependent kinases, cyclins, CDC25C, mitogen-activated protein kinases, Akt signaling, and cell proliferation. Silibinin, whether applied topically or included in the diet, effectively reduced the appearance and development of tumors in the tumorigenesis process. Tumors induced by UVB had higher levels of E2F1 compared to UVB-exposed skin, but silibinin treatment decreased E2F1 levels in the tumors without affecting E2F2 or E2F3 levels. In malignancies, silibinin enhanced apoptosis and increased p53 expression, contrary to its suppressive effect on these factors in UVB-exposed skin cells. The differences observed in the effects of silibinin on E2F1 *versus* E2F2 and E2F3, along with the associated molecular changes and biological consequences in chronically UVB-exposed skin, suggest that silibinin may play a role in interfering with photocarcinogenesis (Yi et al., 2008).

The protective effects of silibinin against UVB-induced epidermal damage were investigated using JB6 cells and SKH1 hairless mice. The results showed that pretreatment with silibinin protected JB6 cells from apoptosis and facilitated the repair of UVB-induced cyclobutane pyrimidine dimers (CPD), which are DNA lesions caused by UVB exposure. Silibinin also reversed the UVB-induced arrest of cells in the S phase of the cell cycle, leading to a decrease in the number of cells actively synthesizing DNA and inactive S phase cells. Mechanistic investigations revealed that UVB irradiation led to a transient increase in phosphorylated and total p53 (a tumor suppressor protein) in the cells. However, pretreatment with silibinin resulted in a sustained upregulation of p53 and its greater localization in the cell nucleus. GADD45, a downstream target of p53 involved in DNA repair and cell cycle control, was also significantly upregulated by silibinin. Importantly, when p53 and GADD45 were knocked out in cells, they became more susceptible to UVB-induced apoptosis without significant S phase arrest, and the protective properties of silibinin were diminished. Furthermore, topical application of silibinin before or immediately after UVB irradiation in SKH1 hairless mice led to increased levels of p53 and GADD45 in the skin epidermis, along with rapid elimination of CPDs. These findings suggest that silibinin’s protective effects against UVB-induced damage are mediated through the p53-GADD45 pathway. Silibinin enhances the expression of p53 and GADD45, leading to improved DNA repair and cell cycle regulation, ultimately reducing the risk of non-melanoma skin cancer (NMSC) and potentially preventing its early onset (Roy et al., 2012).

The effects of silibinin, quercetin, and epigallocatechin 3-gallate (EGCG) on mitogenic signaling and cell cycle regulators were investigated in human epidermoid carcinoma A431 cells, which are commonly used as a model for skin cancer research. Silibinin treatment significantly inhibited the activation of mitogen-activated protein kinase-extracellular signal-regulated kinase-1 and -2 (MAPK-ERK1/2) pathway. It also led to an increase in the levels of Cip1/p21 and Kip1/p27, which are cell cycle inhibitors, and a substantial reduction in the levels of cyclin-dependent kinase 4 (CDK4), CDK2, and cyclin D1, which are cell cycle regulators involved in promoting cell proliferation. On the other hand, treatment with quercetin resulted in a modest increase in Cip1/p21 levels, no change in Kip1/p27 levels, and a decrease in CDK4 and cyclin D1. EGCG treatment induced the expression of Cip1/p21, but there were no changes observed in Kip1/p27, CDK2, or cyclin D1 levels, and CDK4 was reduced only at low levels. All three compounds inhibited cell proliferation in a dose- and time-dependent manner. High doses of silibinin, as well as low and high doses of quercetin and EGCG, induced cell death through apoptosis. It was suggested that the lack of inhibitory effect on MAPK-ERK1/2 activation by these compounds may trigger an apoptotic response associated with their cancer-preventive and anticarcinogenic effects. These findings demonstrate that silibinin, quercetin, and EGCG have distinct effects on mitogenic signaling pathways and cell cycle regulators in A431 cells, which could contribute to their cancer-preventive properties. Understanding these molecular mechanisms can provide insights into the potential use of these compounds as chemopreventive agents for skin cancer (Bhatia et al., 2001).

Silibinin has shown potential in the treatment and prevention of non-melanoma skin cancer (NMSC) in preclinical trials. Over the past 2 decades, silibinin has demonstrated significant potential in combating various malignancies, including NMSCs. One of the ways silibinin protects against NMSC is by preventing UVB-induced thymine dimer production, which can lead to DNA damage. Silibinin increases the levels of p53, a protein involved in DNA repair and cell death, thus promoting the repair or elimination of UVB-damaged cells. Additionally, silibinin targets abnormal signaling pathways and induces anti-inflammatory responses, which have been effective against NMSCs. It has multiple targets within cells, allowing it to defend against cytotoxic agents like reactive oxygen species and inflammation (Prasad et al., 2020).

Silibinin also modulates mitogenic and survival signaling pathways and regulates cell cycle regulatory molecules such as Cip1/p21, contributing to its preventive effects against skin cancer. Studies in mouse models have shown that silibinin has a beneficial effect on repairing UVB-induced DNA damage in the skin. Overall, both in animal models and cell culture experiments, silibinin has demonstrated promising preventive effectiveness against skin cancer. These findings support the notion that silibinin may be a valuable option for the prevention and control of NMSC in humans (Singh and Agarwal, 2005).

### 2.14 Mechanism of action of silibinin in salivary gland cancer

Silibinin has been found to inhibit the growth and metastasis of a human high metastasis cell line of salivary gland adenoid cystic carcinoma (ACC-M) through the activation of autophagy. *In vitro* experiments showed that silibinin dose-dependently and time-dependently inhibited the proliferation of ACC-M cells. Furthermore, silibinin treatment led to an increase in autophagic bodies within the ACC-M cells. Silibinin also demonstrated the ability to enhance the expression of LC3 (microtubule-associated protein 1A/1B-light chain 3), a marker of autophagy, and promote the conversion of LC3-I to LC3-II in ACC-M cells in a dose- and time-dependent manner. LC3-II is specifically associated with autophagosome formation. In an ACC lung metastasis model, the group treated with silibinin showed significantly lower lung weight and fewer left and right lung nodules compared to the control group. Immunohistochemical analysis revealed a significantly higher expression rate of LC3 in the silibinin-treated group. Additionally, the expression levels of both LC3-I and LC3-II were dramatically increased in the silibinin-treated group, while the positive expression of LC3 in human ACC decreased significantly. These findings suggest that silibinin inhibits the growth and lung metastasis of ACC-M cells, potentially through the activation of autophagy. Silibin-induced autophagy may play a role in suppressing tumor progression and metastasis in salivary gland adenoid cystic carcinoma (Jiang et al., 2016).

## 3 Conclusion and future perspectives

**FIGURE 5:**
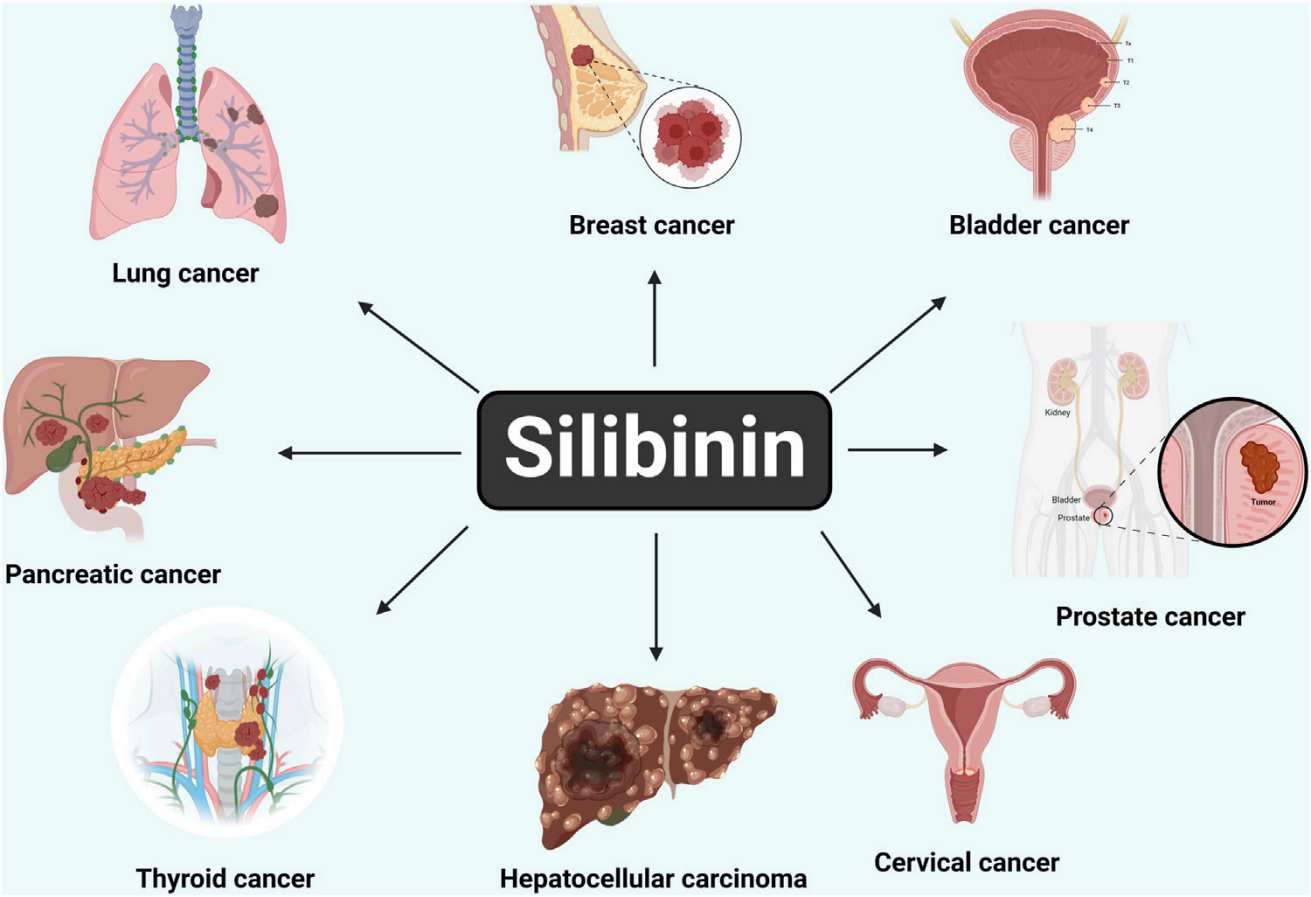
The schematic overview of the review studies.

The findings suggest that silibinin exhibits potent anticancer activity against several types of epithelial cancers, including prostate, breast, skin, colon, lung, kidney, and bladder cancers (Figure 5). It not only acts as an anticancer agent but also possesses important antioxidant properties that contribute to maintaining the body’s homeostasis. One of the unique properties of silibinin is its ability to specifically inhibit the proliferation of tumor cells and suppress abnormal growth in cancer cells. It achieves this by interfering with the function of DNA. When specific regions of the DNA are damaged, it can lead to uncontrolled cell growth, which is a precursor to cancer. Silibinin has been shown to repair these damaged DNA segments, effectively halting abnormal cell growth. In addition to *in vitro* studies, silibinin has been extensively evaluated in *in vivo* studies using mouse and human cancer models, showing promising results in terms of reducing tumor formation and prolonging survival. Ongoing research efforts are focused on developing various drug delivery systems to enhance the pharmacological, pharmacokinetic, and pharmacodynamic properties of silibinin. These advancements aim to improve the efficacy and bioavailability of silibinin as an anticancer agent. Based on the findings of this review, silibinin holds promise as a potential candidate for the development of anticancer drugs. Further research and development are necessary to fully harness its therapeutic potential and optimize its use in cancer treatment.

Indeed, while numerous studies have explored the potential preventive and therapeutic benefits of silibinin in distinct types of cancer, there is still a need for a comprehensive understanding of its precise anticancer mechanisms. Investigating the metabolism of silibinin and identifying its circulating metabolites is crucial for gaining insights into its fate and activity within the body. By characterizing the metabolites of silibinin, researchers can establish a clearer understanding of the relationship between the overall bioactivity of silibinin and the bioactivity of its individual constituents present in target tissues. This knowledge is essential for optimizing the dosage and administration of silibinin in chemoprevention and chemotherapy strategies.

Further research is warranted to delve deeper into the anticancer properties of silibinin and to identify the most effective mode of administration for specific types of cancer. Additionally, efforts can be made to develop pre-formulation and formulation techniques for silibinin, which can facilitate its transition from preclinical studies to clinical research. In summary, more research is needed to enhance the understanding of anticancer properties, determine safe and effective dosages, explore optimal routes of administration, and develop appropriate formulations of silibinin. These endeavors will contribute to harnessing the full potential of silibinin in cancer prevention and treatment.
